# Framework for Evaluating the Health Impact of the Scale-Up of Malaria Control Interventions on All-Cause Child Mortality in Sub-Saharan Africa

**DOI:** 10.4269/ajtmh.15-0363

**Published:** 2017-09-27

**Authors:** Yazoume Yé, Thomas P. Eisele, Erin Eckert, Eline Korenromp, Jui A. Shah, Christine L. Hershey, Elizabeth Ivanovich, Holly Newby, Liliana Carvajal-Velez, Michael Lynch, Ryuichi Komatsu, Richard E. Cibulskis, Zhuzhi Moore, Achuyt Bhattarai

**Affiliations:** 1 MEASURE Evaluation, ICF, Rockville, Maryland;; 2 Center for Applied Malaria Research and Evaluation, Tulane University School of Public Health and Tropical Medicine, New Orleans, Louisiana;; 3 President's Malaria Initiative, Bureau for Global Health, United States Agency for International Development, Washington, District of Columbia;; 4 Avenir Health, Geneva, Switzerland;; 5 Department of Public Health, Erasmus MC, University Medical Center, Rotterdam, The Netherlands;; 6 United Nations Foundation, Washington, District of Columbia;; 7 Independent Consultant based on Stockholm, Sweden;; 8 Division of Data, Research, and Policy, Data and Analytics Section, United Nations Children's Fund, New York, New York;; 9 President's Malaria Initiative, Malaria Branch, Division of Parasitic Diseases and Malaria, Centers for Disease Control and Prevention, Atlanta, Georgia;; 10 The Global Fund to Fight AIDS, Tuberculosis and Malaria, Geneva, Switzerland;; 11 Global Malaria Programme, World Health Organization, Geneva, Switzerland;; 12 IBTCI, Vienna, Virginia;

## Abstract

Concerted efforts from national and international partners have scaled up malaria control interventions, including insecticide-treated nets, indoor residual spraying, diagnostics, prompt and effective treatment of malaria cases, and intermittent preventive treatment during pregnancy in sub-Saharan Africa (SSA). This scale-up warrants an assessment of its health impact to guide future efforts and investments; however, measuring malaria-specific mortality and the overall impact of malaria control interventions remains challenging. In 2007, Roll Back Malaria's Monitoring and Evaluation Reference Group proposed a theoretical framework for evaluating the impact of full-coverage malaria control interventions on morbidity and mortality in high-burden SSA countries. Recently, several evaluations have contributed new ideas and lessons to strengthen this plausibility design. This paper harnesses that new evaluation experience to expand the framework, with additional features, such as stratification, to examine subgroups most likely to experience improvement if control programs are working; the use of a national platform framework; and analysis of complete birth histories from national household surveys. The refined framework has shown that, despite persisting data challenges, combining multiple sources of data, considering potential contributions from both fundamental and proximate contextual factors, and conducting subnational analyses allows identification of the plausible contributions of malaria control interventions on malaria morbidity and mortality.

## INTRODUCTION

Malaria remains a public heath challenge in sub-Saharan Africa (SSA) despite concerted efforts from national and international partners to scale up effective malaria control interventions, such as insecticide-treated nets (ITNs), indoor residual spraying (IRS), diagnostics, prompt and effective treatment of malaria cases, and intermittent preventive treatment during pregnancy (IPTp). The World Health Organization (WHO) estimates that between 2001 and 2013, 670 million fewer cases and 4.3 million fewer malaria deaths occurred globally than would have occurred if incidence and mortality rates had remained unchanged since 2000. Of the estimated 4.3 million deaths averted between 2001 and 2013, 3.9 million, or 92%, were among children under 5 years of age in SSA.^1^

Global investments in malaria control and elimination efforts exceeded USD $2.7 billion in 2013,^1^ resulting in a strong desire from funding partners and national malaria control programs for rigorous evaluations of scaling up malaria control interventions to quantify the impact on malaria-associated morbidity and mortality. Several challenges complicate this task. First, nearly all malaria control interventions are either implemented as national coverage programs or targeted to high-transmission risk areas.^2,3^ In such cases, a contemporaneous control group, or counterfactual, is often unavailable, hindering direct causal inference between exposure to malaria control interventions and any observed changes in malaria health outcomes.^4^ Second, data on malaria morbidity, mortality, and transmission are often unavailable at the required scale or are inappropriate for use in analysis. Third, other factors can affect malaria health outcomes, such as additional maternal and child health interventions, socioeconomic conditions, and environmental factors, but they may not be well documented. Fourth, it is difficult to define an evaluation's baseline, midline, and endline,^4,5^ so that intervention scale-up timing and coverage level obtained must be carefully considered to ensure a complete assessment. Finally, the efficacy of malaria interventions is already proven, unlike other development fields, the evaluation question is more about program implementation of proven interventions and the effectiveness.

A rigorous and valid evaluation design must address these constraints; therefore, in 2007 Roll Back Malaria's (RBM) Monitoring and Evaluation Reference Group (MERG) proposed a framework for evaluating full-coverage malaria control intervention impacts on morbidity and mortality in high-burden SSA countries,^6^ which was the first attempt to propose an evaluation method that fits malaria-control intervention. That framework, however, was theoretical and not yet implemented to uncover potential challenges.

Because standard academic definitions of *evaluation* attribute change in impact measures directly to program interventions, the framework is not, in the strictest sense, an impact evaluation. It is more about plausibility design as opposed to adequacy and probability design.^7^ In this article, impact evaluation refers to the potential contribution of a package of malaria control interventions, which could be national malaria programs, to change malaria morbidity and mortality (Table 1, definition of key terms).

**Table 1:** Definition of key terms used in this paper

Terms	Definition
All-cause child mortality rate	Probability of dying from any cause between the first and fifth birthday per 1,000 children who survived to age 12 months^17^
Civil registration and vital statistics	A system for recording vital events in a population, including births and deaths, with medical certification of the cause of death according to the rules and procedures of the International Classification of Diseases
Confirmed malaria case	Suspected malaria case in which malaria parasites have been demonstrated in a patient's blood by microscopy or a rapid diagnostic test^32^
Contextual factors	Non-malaria programs and other factors, such as rainfall, socioeconomic status, urbanization, and policy changes, that could confound the association between scale-up of the intervention and its potential health impact or modify the effect of the intervention, and affect the conclusion
Impact evaluation	Within the context of this paper, impact evaluation refers to the potential contribution of a package of malaria control interventions, which could be considered the national malaria program, to a given outcome
Malaria parasitemia	Presence of malaria parasites in the blood or number of parasites per volume of blood
Malaria parasite prevalence	Proportion of children ages 6–59 months with malaria parasite infection^17^
Malaria transmission	Spread of malaria by completion of a full transmission cycle (man→mosquito→human)
Malaria transmission intensity (force of infection)	Measured as entomological inoculation rate (EIR): the number of infectious mosquito bites a person is exposed to in a certain time period, typically a year
Malaria-specific mortality	Deaths in which malaria was the underlying cause. The World Health Organization (1993) defines it as “the disease or injury which initiated the train of morbid events leading directly to death”
Plausibility argument	An assumption that mortality reductions can be attributed to programs if improvements are found along the causal pathway between intervention scale-up and mortality trends^7^
Population-level malaria morbidity indicators	Indicators on malaria morbidity collected through population-based surveys; examples are malaria parasite prevalence and anemia
Under-five mortality	Probability of dying before the fifth birthday per 1,000 live births
Verbal autopsy	A method for determining cause of death. A knowledgeable person in the household where a deceased person lived is asked about signs and symptoms of the terminal illness, usually 1–6 months after the death.^18,22,28^ To attribute causes of deaths, interviews are analyzed by an algorithm or clinicians who decide on causes by majority vote^6^

This article expands the RBM/MERG framework and highlights some of the challenges in applying this theoretical framework. In addition, we take the RBM/MERG framework further by including secondary analyses to more rigorously examine causal inference. These analyses include stratification, use of a national-platform analytic framework, and analysis of birth history data from national household surveys to assess child survival. Our framework takes into account new program data from intervention scale-ups since 2007 and new evaluation experience.^8,9^ This updated framework may be useful to stakeholders that are responsible for implementing and evaluating malaria intervention impacts, such as ministries of health, national malaria control programs, and development partners that support malaria control efforts.

## MATERIALS AND METHODS

### Evaluation objectives.

The main objectives of an malaria control intervention impact evaluations are to 1) measure the degree of implementation success and outcomes compared with targets set in the national strategic plan, 2) assess trends in malaria-related morbidity and all-cause under-five mortality as a complementary malaria impact indicator, and 3) assess the plausible attribution of the malaria control intervention scale-up on malaria-related morbidity and all-cause under-five mortality, and also account for contextual factors, such as determinants of malaria transmission and child survival.

Following are the specific evaluation questions:

- Was the scale-up sufficient enough to affect national-level malaria-specific morbidity and all-cause under-five mortality?
- Was sustained coverage of malaria control intervention achieved? Was the coverage equitable across socioeconomic groups?
- Was the increase in coverage of interventions associated with impact on national all-cause under-five mortality?
- Was impact highest among those risk groups with the greatest potential to benefit from exposure to the interventions? And was the impact equitable across socioeconomic groups?
- Can other plausible explanations of the observed change in national all-cause under-five mortality be excluded?

### Evaluation design.

An experimental study design is assumed to be the gold standard for assessing efficacy of new interventions.^5^ Using such a design to evaluate a package of malaria control interventions is challenging for several reasons, the foremost being a lack of randomization to designate an intervention group and a control group, or a counterfactual. Where malaria control interventions are scaled up or intensified staggered over time, it may be possible to use a step-wedge quasi-experimental evaluation design.^7,10,11^ However, the step-wedge design is subject to variations among wedges regarding geographical settings, population, or transmission patterns. The design is also subject to contamination among wedges, such as the higher burden areas may receive interventions first, compared with those with lower burden, and randomizing between the two settings poses significant ethical challenges. Also, limited subnational-level longitudinal, population-based data on the coverage of malaria control interventions and health outcomes precludes evaluation designs, such as a national platform approach, to assess a dose-response relationship between coverage and outcomes.^11^

**Figure 1.**
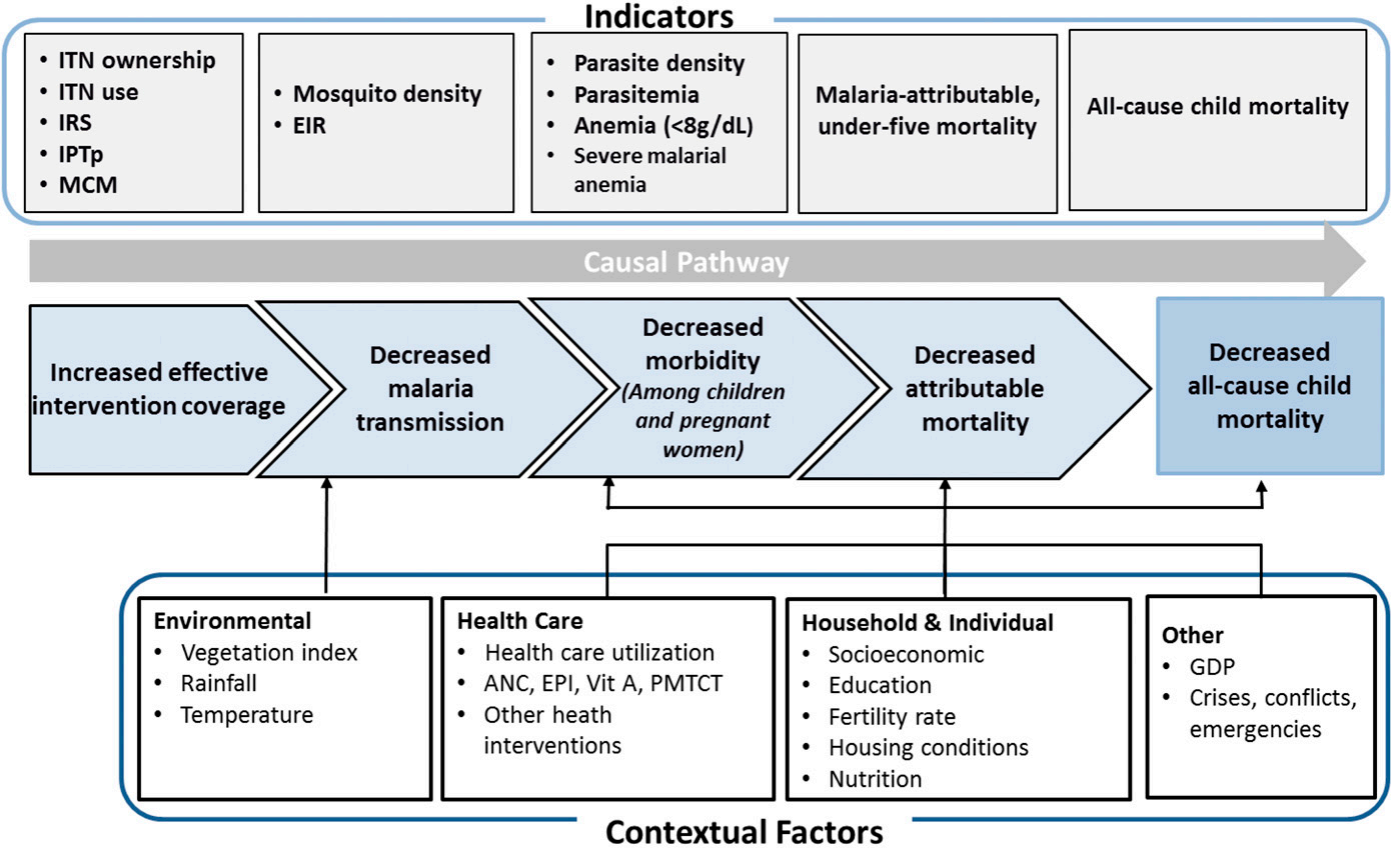
Plausibility study design framework for assessing malaria control intervention impact on malaria morbidity and all-cause child mortality. ANC = antenatal care; EIR = entomological inoculation rate; EPI = extended program for immunization; ITN = insecticide-treated net; IRS = indoor residual spraying; IPTp = intermittent preventive treatment; GDP = gross domestic product; MCM = malaria case management; Vit = vitamin; PMTCT = prevention of mother to child transmission. This figure appears in color at www.ajtmh.org.

To address these constraints, a prepost reflexive control study design, also known as the ecological or plausibility study design, for evaluating the effectiveness of national-coverage malaria control interventions has been proposed.^6,7,11^ The plausibility study design contends that if the interventions have already proven efficacious on malaria outcomes, then it is plausible that their scale-up could have contributed to or driven the observed reductions in malaria burden, unless other contextual factors and nonmalaria related interventions are unlikely to explain all of these reductions in burden. For example, randomized controlled trials have established protective efficacy of ITN use on the incidence of malaria illness and all-cause under-five mortality;^12–14^ therefore, increasing coverage of ITNs use should reduce malaria morbidity and mortality over a time period consistent with the impacts established in the trials. The plausibility design is strengthened if malaria burden baseline levels can be established before intervention scale-up, with the counterfactual being that pre-scale-up malaria trends would have continued in the absence of the interventions. Thus, over time, the intervention group serves as its own comparison group (Figure 1).

The strongest plausibility assessment can be made when there is evidence of a statistically significant increase in intervention coverage and use that is substantial enough to achieve a realistically proportional reduction in malaria-related morbidity and all-cause under-five mortality within the time frame expected (Table 2). The relationships between increasing coverage and use and reducing morbidity and mortality; however, are not linear. The plausibility assessment must also document contextual factors, both health-related and not, that may affect changes in malaria-related morbidity and child mortality.

**Table 2:** Plausibility study design strengths, limitations, and assumptions

Strengths	Limitations	Key assumptions
Intervention group serves as its own control over time	No true counterfactual, so cause and effect cannot be conclusively inferred	Program is preexisting or full (above threshold) coverage
No need to exclude any population or group from the intervention/program, so can be applied to programs with nation-wide coverage	Multiple sources of data, analyses, and triangulations needed to establish plausible impact	Pretest (baseline) data for the relevant indicators can serve as counterfactual scenario
	Differential selection bias and attrition risk to cause bias and dilution of impact	No other plausible explanations for observed outcomes or any likely confounder effects can be adjusted for
Can adapt to use existing data collected for other purposes (DHS, MICS)	Data might not be as specific as required	All-cause under-five mortality is a sensitive, specific, and time-sensitive proxy for changes in malaria-specific mortality in highly endemic countries

This plausibility assessment assumes evaluators can 1) reliably measure changes in malaria intervention coverage, malaria health outcomes, and contextual factors over time; 2) determine that increases in malaria intervention coverage adequately preceded changes in malaria health outcomes; and 3) record whether health impact was highest among specific risk groups with the greatest potential to benefit from exposure to the interventions. The plausibility design should take into consideration all of the Bradford Hill criteria for causality, as outlined in Table 3.

**Table 3:** Bradford Hill causality criteria, as applied to plausibility assessment

Criterion	Description	Assumptions
Strength of association	Strong associations are more likely to have causal components than weaker associations.	Associations can be measured
Consistency	Observing similar evaluation results across evaluation methods, over time, and across countries from meta-analyses increases the likelihood of causal relationships.	Results have been measure consistently over time and space
Specificity	Observing an association specific to outcomes of interest among specific groups increases the argument for causal effect.	Malaria interventions are highly likely to reduce all-cause under-five mortality, particularly among vulnerable groups
Temporality	Changes in program must precede changes in disease or coverage outcomes.	Scale-up of interventions has been measured
Gradient	Changes in disease or coverage outcomes increase the same amount for increases to program exposure or intensity.	Coverage has been measured in different geographic areas
Plausibility	Biological plausibility links exposure to intervention with health outcome.	Malaria contributes to all-cause child mortality
Coherence	Causal inference is possible only if the literature or substantive knowledge supports this conclusion	There are documented studies showing that malaria interventions affect mortality
Experiment	Causation is a valid conclusion if researchers have seen observed associations in prior experimental studies.	There are documented studies showing that malaria interventions affect mortality
Analogy	For similar programs operating, similar results can be expected to bolster the causal inference concluded.	Program context has been similar in the past

### Evaluation timing.

**Figure 2.**
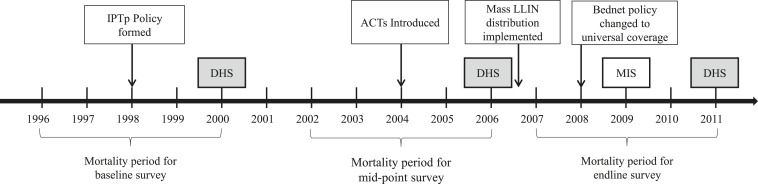
Example evaluation timeframe, from Evaluation of the Impact of Malaria Control Interventions on All-Cause Mortality in Children Under-Five in Uganda. Source: Unpublished report). DHS = Demographic and Health Surveys; MIS = Malaria Indicator Survey; ACT = artemisinin-based combination therapies; LLIN = long-lasting insecticidal nets; IPTp = intermittent preventive treatment of pregnant women.

Strategically timing an impact evaluation is important in achieving the most effective, informative results. Stakeholders should be enlisted to develop criteria to define the evaluation baseline, midline, and end line, based on when malaria control intervention implementation occurred and the availability of data points.^6^ When possible, baseline outcome measures should be established well before malaria control intervention scale-up because under-five mortality estimates from household surveys reflect the situation an average of 2.5 years preceding the survey. Figure 2 illustrates a sample evaluation timeframe, including intervention milestones and timing of data sources.

There is no simple, direct way to determine when an impact evaluation becomes appropriate—the exact time point after malaria control intervention scale-up begins or the level of intervention coverage. If the scale-up is still in the early stages, coverage levels may still be too low, and an impact evaluation may not be recommended; however, it is difficult to state a threshold for when national-level impact is likely to become measurable. Conducting an evaluation takes substantial planning and inputs, as illustrated in the process of RBM impact evaluations led by the President's Malaria Initiative (PMI).^15^ A program performance review that assesses program implementation success may be suitable when impact evaluations are not.^16^

### Evaluation indicators.

#### Changes in coverage of malaria control interventions.

MERG issued a set of primary population-level indicators to measure changes in coverage of key malaria control interventions.^17^ These indicators measure how well malaria control interventions are being implemented to meet coverage targets defined in national malaria control strategies.^3^ Key indicators include ITN ownership and use, households protected by IRS, pregnant women receiving IPTp, and children receiving effective treatment of malaria (Table 4).

**Table 4:** Key primary outcome indicators used to assess malaria control intervention scale-up

Indicator	Purpose/rationale of indicator
Vector control	
Proportion of households with at least one ITN	Measures household ITN ownership
Proportion of households with at least one ITN for every two people	Measures the proportion of households with sufficient ITNs to cover all individuals who spent the previous night in surveyed households, assuming an average of two people sharing each ITN
Proportion of population with access to an ITN in their household	Measures the proportion of the population that could have slept under an ITN, assuming each ITN is used by two people
Proportion of the population that slept under an ITN the previous night	Measures the level of ITN use among all individuals who spent the previous night in surveyed households, regardless of whether those individuals had access to an ITN in their household
Proportion of children under 5 years old who slept under an ITN the previous night	Measures the level of ITN use of children under 5 years old.
Proportion of pregnant women who slept under an ITN the previous night	Measures the level of ITN use among pregnant women
Proportion of existing ITNs used the previous night	Measures the use of existing ITNs. In certain instances, calculating the proportion of existing ITNs used the previous night will be useful for assessing the utilization of existing ITNs and determining the magnitude of nonuse of ITNs at the time of the survey
Households covered by vector control: proportion of households with at least one ITN and/or sprayed by IRS in the last 12 months	Measures the proportion of household protected by an ITN or IRS
Universal coverage of vector control: proportion of households with at least one ITN for every two people or sprayed by IRS within the last 12 months	Measures progress towards achievement of universal coverage of malaria prevention through the two main vector control activities
Intermittent preventive treatment during pregnancy (IPTp)
Proportion of women who received three or more doses of IPTp for malaria during antenatal care visits during their last pregnancy	Measures national level coverage of use of IPTp to prevent malaria during pregnancy among women who gave birth in the last two years.
Case management	
Proportion of children under 5 years old with fever in the last 2 weeks who had a finger or heel stick	Measures national-level coverage of parasitological diagnosis among children under 5 years of age
Proportion of children under 5 years old with fever in the last 2 weeks for whom advice or treatment was sought from a formal health-care provider	Measures national-level coverage of health seeking behavior for malaria from the formal health care providers among children under 5 years
Proportion of children under 5 years old with fever in last 2 weeks who received first-line antimalarial treatment according to national policy	Measures national-level treatment coverage of children under 5 years are in accordance with national first-line malaria treatment policy.
Proportion receiving treatment with recommended first-line antimalarial, among children under 5 years old with fever in the last 2 weeks who received any antimalarial drugs	Measures what proportion of antimalarial treatment received by children under 5 years are in accordance with national first-line malaria treatment policy.

Source: Adapted from Roll Back Malaria, 2013, *Household Survey Indicators for Malaria Control*. ITN = insecticide-treated net; IRS = indoor residual spraying; IPTp = intermittent preventive treatment of pregnant women.

Primary data sources for population-level coverage include national household surveys, such as the Demographic and Health Surveys (DHS), Malaria Indicator Surveys (MIS), and Multiple Indicator Cluster Surveys (MICS). These surveys typically are conducted every 3–5 years and provide national and subnational estimates for intervention coverage indicators. Further coverage data may come from health management information systems (HMIS), Health and Demographic Surveillance Systems,^18^ or special surveys conducted for specific projects, such as post-campaign long-lasting insecticidal nets coverage surveys. Other malaria program data, such as timing and quantity of malaria commodity distribution, might also help understand intervention coverage. Data from these sources, with the potential to contribute to measuring and understanding coverage, should be evaluated for rigor and incorporated in the analysis when appropriate, considering scope, sampling, and the data collection method.

#### Malaria morbidity, mortality, and transmission indicators.

Indicators of key malaria outcomes are used to assess how the burden of malaria has changed over time, presumably as a result of malaria control intervention scale-up. Examples of these outcomes include malaria morbidity, determined by prevalence of malaria antigens or parasites; density of parasitemia (parasites/μL) or severe anemia (< 8 g/dL) in children under 5 years of age,^19^ or all-cause under-five mortality. When data are available, an outcome indicator can be determined from malaria transmission intensity, measured through malaria infection incidence rates, or force of infection, or the entomological inoculation rate.^20,21^

Health Management and Information Systems have the potential to provide malaria-specific morbidity and mortality data, when proper diagnosis and medical certification of cause of death are available. The quality of the data depends on consistent, appropriate use of diagnostics and inpatient and outpatient case management. A change in diagnostic methods over the evaluation period, such as purely clinical or microscopy or rapid diagnostic test, can affect morbidity trends. In addition, health-care utilization trends and reporting rates can affect malaria morbidity and mortality calculations for health facilities. Because health-care utilization rates are low in most SSA malaria-endemic countries, only a potentially time-varying fraction of malaria deaths are likely to occur at public health facilities. Many malaria-related deaths occur at homes or at private facilities that do not link into HMIS.^1^

Verbal autopsy is so far the only available and accessible method to measure malaria-specific mortality at the population level in a context where most deaths occur outside a formal health facilities. With verbal autopsy, the deceased's primary caregivers are interviewed for detailed information about events leading up to the death to ascertain and assign a cause.^22–25^ Existing verbal autopsy tools are not highly sensitive,^26^ but efforts are underway to increase sensitivity in detecting cause of death, such as using computer-based algorithms and the simplified symptom pattern approach.^27^ Although verbal autopsy may not provide a precise measure of malaria-specific mortality, it may give an idea of the ranking of malaria in a list of causes of death at to population level and help understand further malaria epidemiology.^28^ In countries where such data exist, evaluations should consider exploring verbal autopsy for use in the plausibility assessment, particularly as a case study because the information in most cases is not available at the national level.

Civil registration and vital statistics systems are also potential data sources for measurement of population-level cause specific mortality.^29^ However, in most countries data are not strong enough to use in impact evaluations at the national level,^30^ a circumstance that could change as confirmation of malaria diagnosis with parasite density becomes more routine and medical autopsies are performed.^18^

In the absence of reliable malaria-specific mortality data, and given the large indirect effects that malaria control interventions have on child health,^31^ RBM and WHO recommend using all-cause under-five mortality as the standard impact measure in malaria-endemic countries.^17,31,32,33^ This is commonly measured through standardized national household surveys, such as DHS and MICS, in most malaria-endemic countries.

#### Contextual factors.

Contextual factors that may influence outcome or impact indicators should be investigated for their potential contribution to changes in the outcomes of interest. Where possible, these contextual factors should be directly accounted for in the analytic framework used to assess the association between changes in malaria control intervention coverage and malaria health outcomes. Contextual factors can be divided into two groups, 1) distal (e.g., gross domestic product, housing condition, maternal education, and climate) and 2) proximal (e.g., nutritional status, childhood vaccination coverage, vitamin A supplementation, and breastfeeding). Fluctuation in contextual factors is expected, so evaluators should only look for anomalies that may explain changes in all-cause under-five mortality, within the given the timeframe (Table 5).

**Table 5:** Examples of contextual factors that should be examined

Category	Examples	Data sources	Justification
Child survival interventions	Expanded program on immunization coverage, such as measles and DPT3,	WHO, UNICEF annual estimates of national immunization coverage	Observed reductions in child morbidity and mortality may actually be result of increased coverage of these programs rather than malaria control interventions.
	micronutrient supplementation coverage, including vitamin A, iron, and zinc	UNICEF vitamin A coverage database	
		DHS, MICS, MIS	
Climatic and environmental factors	Total precipitation	National meteorological agency	These factors affect mosquito breeding and malaria transmission and may cause observed changes in outcomes over time or geography, rather than the interventions themselves.
	Number of days with rain	Columbia University Earth Institute climate database	
	Land cover and vegetation	National Oceanographic and Atmospheric Administration	
	Air temperature		
	Extreme weather events, such as floods		
Health systems factors	Per capita expenditure on health	WHO/WHOSIS	Health systems can affect comparisons across time or geography by influencing access to interventions. These factors modify the impact of malaria control interventions.
	Government expenditure on health as percentage of total government expenditure	The World Bank development indicator database	
	Availability of essential drugs		
	Political situation and stability		
Socioeconomic factors	Household assets and income	DHS, MICS	If different socioeconomic groups access malaria control interventions differently, these factors may serve as effect modifier influence outcomes.
	Parental education	The World Bank development indicator database	
	Conflict or emergency settings		
	GDP per capita, Gini per capita		
	Population living below poverty line		

DPT3 = diphtheria, pertussis, tetanus vaccine, 3 doses; WHO = World Health Organization; UNICEF = United Nations Children's Fund; DHS = Demographic and Health Surveys; MICS = Multiple Indicator Cluster Surveys; MIS = Malaria Indicator Survey; GDP = gross domestic product.

### Data analysis.

The primary method to build plausibility is to assess trends in malaria intervention coverage, malaria health outcomes, and potential contextual factors. An example is the Zanzibar North A district program evaluation that incorporated data from multiple sources, including HMIS-derived monthly incidence cases as the primary outcome, and explanatory variables, such as the year of program intervention and monthly rainfall from meteorological sources, all analyzed in a negative-binomial regression framework.^34^ In another example from Zanzibar, evaluators used log-linear regression to assess reductions in malaria cases and deaths between the preintervention period of 1999–2003 and 2008.^35^ Evaluators concluded that significant reductions in malaria cases and malaria-related mortality were made during the period evaluated.

Another example is the evaluation of the Bioko Island Malaria Control Project^36^ in Equatorial Guinea that assessed 4 years of high-coverage scale-up of IRS, ITNs, and improved case management on the island. Researchers used survival regression to measure the change in all-cause mortality among children under 5 years of age. Calendar year was included as a covariate to test the presence of a declining trend in child mortality. Household access to electricity was included as a proxy indicator of household wealth. A subsample was used to test the association between rainfall in the previous 12 months and child mortality. Variables of interest included possession and use of ITNs, IRS coverage, and pregnant women protected through IPTp. As hypothesized, results indicated simultaneous drops in the prevalence of malaria parasite infection, anemia, reported fever, and all-cause under-five mortality in children. This evaluation, however, did not include an in-depth discussion of contextual factors that could also have influenced the decline in all-cause under-five mortality.

The RBM impact evaluations, led by PMI, took advantage of a key strategy for data analysis: risk stratification. To strengthen the plausibility design, analyses of parasite prevalence, anemia prevalence, and mortality were stratified by age group, place of residence, and malaria risk zone, where data were available.^8^ The stratification helped identify areas and subpopulations with the greatest potential to benefit from interventions and assess whether these groups experienced the highest reductions in morbidity or mortality during the evaluation. If reduction in anemia is associated with malaria decline, we would expect to see a higher baseline and greater reduction in severe anemia prevalence in areas with relatively higher malaria risk, considering that other factors remain constant.^37^ Furthermore, if a major part of the decline in mortality among children under five was malaria related, we would expect a greater mortality decline among children under five living in areas of greater malaria risk, compared with areas of lower malaria risk, if other factors remain constant.^37,38^ In a similar way, we would expect a greater decline in mortality among the age group most likely to experience malaria-related death (6–24 months), compared with other childhood age groups, if malaria control interventions were the main mortality reduction driver.^39,40^ However, the age groups might change with the reduction of malaria infection. In countries where significant reduction have been achieved, it might be advisable to explore older age groups.

The results from such analyses to assess trends in malaria morbidity and mortality can be strengthened using further analytic methods tailored to the available data in a specific country. Building on previous work using a national-platform approach, where districts or district-time units formed the unit of analysis,^41–43^ an available survey data analysis in Malawi used a Poisson model at the district level to examine the association between ITN coverage and all-cause child mortality.^12^ In Uganda and Senegal, evaluators conducted a regression survival analysis to examine differences in child survival before and after the scale-up began. Separately, the analysis examined trends in variables, such as mother's education, DPT3 coverage, socioeconomic growth, access to health care, and temperature and rainfall anomalies. These types of analyses can be undertaken, depending on data availability and usefulness to the objectives of the evaluation itself.

**Figure 3.**
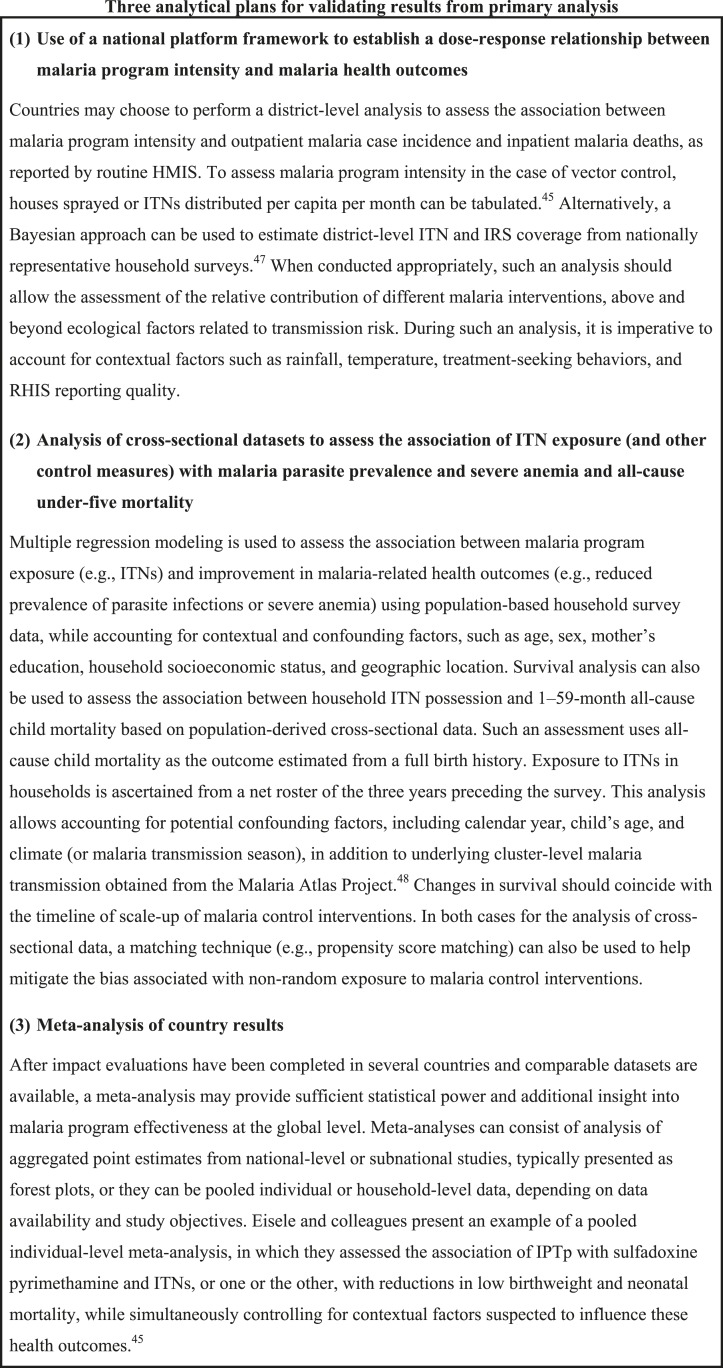
Three analytical plans for validating results from primary analysis.

Data may also be pooled across countries to provide evidence of malaria control intervention effects at the global level (Figure 3).^19,43^ After evaluations have been completed in several countries and comparable datasets are available, this kind of meta-analysis could provide more insight into malaria program effectiveness, especially where in-country analyses lack sufficient statistical power to detect program effects.

## DISCUSSION

A consistent, accurate method is needed to evaluate the impact of the remarkable scale-up of malaria control interventions in SSA. Clear methods have yet to be identified to directly measure the impact of this scale-up, but this paper proposes an enhanced plausibility design.

The ability to attribute impact to interventions depends on the type of design. The gold standard to attribute causality is an experimental design because of the random assignment of units to intervention and control groups; however, this design is challenging for malaria control programs because intervention areas are based on nonrandom considerations and often are implemented at a national scale. Malaria control programs fall under the category of “complex intervention research,”^4^ with multiple interventions occurring simultaneously and interacting. In addition, interventions target various groups and sometimes require beneficiaries to adapt a number of behaviors to achieve the intended impact.

The impact evaluation design of malaria control intervention scale-up falls in the area of developing literature on complex or system-level interventions and plausibility evaluations.^4,44^ This literature suggests that, in addition to measuring changes in key indicators before and after an intervention, an evaluation should include four considerations: 1) a theoretical understanding of how the intervention causes change through an impact model; 2) allowance for variability in outcomes, depending on population characteristics, through the estimation of separate outcomes for different strata; 3) an assessment of the implementation process to determine if any lack of impact reflects implementation failure or genuine ineffectiveness, which can guide and improve program management; and 4) a thorough documentation of context to inform assessments about causality and help generalize results to other contexts.

The improved plausibility design proposed here builds on these principles. The design is advantageous in certain settings because it obviates the need for a control group, and it can assess the impact of national coverage programs. The success of this design, however, requires comprehensive and consistently high-quality data, as well as rigorous analytical techniques. Data from multiple sources in each country should be used to compensate for the shortcoming of any one method of data collection. For example, HMIS often underestimate the total deaths attributed to malaria because most malaria-related deaths in SSA occur outside of public health facilities; however, these data can be combined with confirmed-case reporting to more rigorously assess malaria control intervention impact.^40^ Potential confounders should always be thoroughly investigated in trend analyses and other statistical models to evaluate the role of these factors on the health outcomes of interest.

Although the expanded evaluation framework provides a good basis for evaluating malaria control intervention impact, it does have some limitations. It is still unclear when or at what threshold of intervention coverage an impact evaluation becomes appropriate. The plausibility study design assumes that scale-up and its impact, along with contextual factors, can all be measured reliably at national and subnational levels; however, significant gaps still exist in data acquisition systems in most malaria-endemic countries. The proposed framework relies exclusively on secondary data and its application depends on the quality of the data. Therefore, additional efforts need to be put into strict data collection at the national and subnational levels.

Moreover, baseline data preceding the scale-up of malaria control interventions do not exist for many countries, making it difficult to measure change over time. Countries should continue to invest in strengthening routine health information system to ensure that periodic household surveys are complemented by robust longitudinal data from health facilities where malaria is diagnosed and treated. This is particularly important in changing malaria epidemiological context with countries moving from control to pre-elimination phase. As a starting point, a comprehensive mapping and cataloguing of routine health information systems data elements, data quality and data gaps will be helpful and serve as a baseline for improvement of the systems. Although similar mapping and catalogue exist for household surveys it is lacking for routine health information systems. Furthermore, baseline coverage levels will continue to shift as ongoing scale-up and maintenance of interventions affect malaria transmission and environmental changes alter malaria intensity. This new context will require changes in evaluation approaches and more reliable, flexible, timely, and representative systems for measuring changes in malaria control interventions and health outcomes.

It is also difficult to develop an accurate counterfactual scenario that captures a prescale-up level of intervention coverage and program intensity. If malaria was already in decline before scale-up, then continuation of prescale-up trends is a conservative or pessimistic counterfactual because this scenario must also assume that baseline coverage increases are also continuing. The program's contribution is then only the recent acceleration of coverage increases, without accounting for the past and current scale-up in coverage. On the other hand, if malaria had been on the rise before scale-up, assuming a continuation of that rise as counterfactual results in an optimistic estimation of impact. Malaria is an epidemic disease, and often a rise is followed by a decline, even without program effort. In addition, urbanization and socioeconomic development may be leading to declining secular trends in most countries.

The relationship between malaria coverage and impact has a time lag, and the dynamics of how burden trends respond to coverage trends is highly nonlinear. Malaria interventions may reach their full impact several years after coverage scale-up; however, that impact may include a partial rebound: as interventions reduce cases, people build up less acquired immunity, which can result in older age groups having a final new stabilized burden level that may not be as low as previous burden levels (e.g., the level 2 years after reaching maximum intervention coverage).

Despite all these challenges, the plausibility approach proposed in this paper provides a good avenue to document evidence of malaria control achievements in endemic countries. However, as data become more available and information systems are strengthened, further improvement to this approach will be needed.
